# Comprehensive Effects of Potassium Lactate, Calcium Ascorbate and Magnesium Chloride as Alternative Salts on Physicochemical Properties, Sensory Characteristics and Volatile Compounds in Low-Sodium Marinated Beef

**DOI:** 10.3390/foods13020291

**Published:** 2024-01-17

**Authors:** Shujie Yang, Xiaoli Ma, Yanfeng Huang, Boyue Lin, Longtao Zhang, Song Miao, Baodong Zheng, Kaibo Deng

**Affiliations:** 1 College of Food Science, Fujian Agriculture and Forestry University, Fuzhou 350002, China; starysjj@163.com (S.Y.); mxl025807@163.com (X.M.); hyfeng0606@163.com (Y.H.); lby100551@163.com (B.L.); zlongtao@fafu.edu.cn (L.Z.); zbdfst@163.com (B.Z.); 2 China-Ireland International Cooperation Centre for Food Material Science and Structure Design, Fuzhou 350002, China; song.miao@teagasc.ie; 3 Teagasc Food Research Centre, Food Chemistry and Technology Department, Moorepark, Fermoy, P61 C996 Co. Cork, Ireland

**Keywords:** compound alternative salts, moisture, microstructure, sensory evaluation, volatile flavor compounds

## Abstract

The search for alternative salt formulations similar to sodium chloride and their effect on marinated meat products is of great significance to the low-sodium meat processing industry. The main purpose of this study was to investigate the effect of partially replacing sodium chloride with potassium lactate, calcium ascorbate, and magnesium chloride on the sodium content, water activity and distribution, protein solubility, microstructure, sensory characteristics and volatile flavor compounds in low-sodium marinated beef. The sodium content in the test group decreased up to 28% compared to 100% in the sodium chloride group C1. The formulation including 60% sodium chloride and a total of 40% compound alternative salts in groups F1 and F2 increased their myofibril fragmentation index and promoted the disruption of the myogenic fiber structure. Group F1 (the ratio of potassium lactate, calcium ascorbate and magnesium chloride was 2:1:1) performed higher solubility of myofibrillar proteins and lower transverse relaxation value than group F2 detected by low-field nuclear magnetic resonance, which indicated that F1 formulation was beneficial to promote the solubility of myofibrillar proteins and attenuate the water mobility of marinated beef. Moreover, group F1 had a more similar microstructure and more similar overall sensory attributes to group C1 according to the scanning electron microscopy. The sensory evaluation showed higher peak intensity and response values of volatile flavor compounds than group C1 and C2 (only 60% sodium chloride) when detected using gas chromatography–ion mobility spectrometry technology, which indicated that the compound alternative salts of group F1 can improve the lower quality of low-sodium marinated beef and perform similar attributes to the C1 sample regarding moisture distribution and microstructure and even performs better than it with regards to flavor. Therefore, the F1 formula possessed greater potential for application in low-sodium marinated meat products.

## 1. Introduction

Salt plays an indispensable role in the curing process of meat products. On the one hand, salt has a good antiseptic effect, which can reduce the water activity of food, inhibit the growth of microorganisms and prolong the shelf life of food [1]. On the other hand, the penetration and distribution of salt changes the protein state and tissue structure in muscle, including promoting dissolution of myofibrillar protein and increasing the hydration capacity of protein [2]. In addition, salt is also regarded as a good flavor enhancer, which can catalyze the reaction process of taste substances and flavor substances in muscles, and enhance the flavor of meat products [3,4].

Raw meat is usually low in sodium, but after commercial processing into meat products, the sodium content rises significantly, with some marinated meat products adding up to 5% [5]. The meaning of marinated meat products varies widely between countries [6], and that of a Chinese-type is always referring to flavoring of the meat by marination ingredients or additives, which is also what is stated in Chinese domestic trade industry standard [7]. Sodium is an essential nutrient to maintain the normal physiological functions of the human body and has various functions such as regulating the osmotic pressure balance and water metabolism of the human body [8]. However, excessive sodium intake will increase blood pressure, leading to a rising incidence of chronic cardiovascular disease, as well as a series of diseases such as gastric cancer, hypertension and osteoporosis [5,9]. Therefore, the process of sodium content reduction in the food industry is in line with consumer concerns about nutrition and health [10].

Excessive regulation of salt content can induce unpalatability in meat products; therefore, sodium chloride replacers must be added to meat products to reduce the sodium content. The alternative salts commonly include potassium, calcium and magnesium salts [11,12,13,14]. From a health point of view, the intake of potassium, calcium and magnesium in a certain dose can also make up for the lack of calcium and magnesium intake in the human body’s daily diet. Studies have found that there is a synergistic effect between potassium lactate and sodium chloride, which can increase the salty taste, improve the palatability of meat, and keep the color of the product stable [15,16]. With the increase in potassium lactate from 0% to 2% in Rugao ham, the water became strongly combined and the sensory scores increased [16]. In addition, it can also promote the dissolution of Z-line in muscle, improve water retention, increase the yield, and inhibit the growth of microorganisms to ensure product safety [17]. Potassium salt is regarded as the most common single alternative salt; however, excessive addition of it always induces an unpleasant metallic and bitter taste in meat [18]. Magnesium chloride possesses the ability to promote myofibrils dissolution, improve protein emulsification, and reduce juice loss [19], but these effects are not so significant in the presence of potassium, which may be attributed to its divalent nature leading to a relatively weakened cross-linking effect of protein [20], so it can also replace a small amount of sodium chloride. Calcium ascorbate is used as an antioxidant and preservative in processed meat products, which can affect the redox ability of meat products, help meat products to form a stable color and prolong the shelf life of food [21]. However, when only calcium ascorbate was used to partially substitute sodium salt, it still resulted in a significantly greater difference in dry-cured hum compared to the full sodium group [22]. Due to the excessive substitution of sodium chloride by a single salt, the organoleptic texture, and flavor of meat products may be adversely affected. Therefore, compounding with these alternative salts is a good way to reduce the sodium chloride content while maintaining the quality of meat products [23].

Effects of these alternative salts on marinated meat products in present research is still scanty and the research mainly focuses on the flavor attributes of dry-cured and fermented meat products [3,24,25]. We aimed to identify a feasible alternative salt formulation for the low-sodium marinated beef slice process and determine its impact on more specific quality attributes. So, the overall purpose of this study was to analyze the effects of three alternative salts, potassium lactate, calcium ascorbate, and magnesium chloride, on moisture distribution, protein structure, sensory characteristics and volatile compounds in low-sodium marinated beef.

## 2. Materials and Methods

### 2.1. Materials

The chilled beef was purchased from a local supermarket and transported to the laboratory within one hour. Sodium chloride, potassium lactate, calcium ascorbate and magnesium chloride were all food graded (Shanghai Jinjing Chemical-engineering Co., Ltd., Shanghai, China). All chemicals were purchased from Solarbio Corporation (Beijing, China).

### 2.2. Marinated Beef Preparation

Chilled beef knuckle without visible fat and connective tissues was cut into 30 mm × 20 mm × 8 mm meat slices along the muscle fiber direction, and vacuum-packed and stored at −18 °C. Before pickling, the samples were thawed at 4 °C for 12 h, randomly divided into 4 groups for pickling in different pickling solution (100 g samples in each group) and statically immersed into 200 mL pickling liquids for 4 h at 4 °C. The pickling solution in control group C1 contained 4% sodium chloride. Another control group, C2, was carried out using the same pickling solution but only half the content of sodium chloride. Based on equal mass substitution, the sodium chloride content of the C1 group was regarded as 100%, and different proportions of potassium lactate (20%), calcium ascorbate (10% and 15%) and magnesium chloride (5% and 10%) were used to replace the sodium chloride, which were named as group F1 and F2 shown in Table 1, respectively. After marinating, the meat slices were taken out, the surfaces were rinsed with distilled water, the water was absorbed with a tissue gently, and then the samples were taken for relevant indexes determination. All percentages in this study represented mass percent.

### 2.3. Sodium Content

The sodium content was determined according to the method described by Triki et al. [26]. The marinated beef was ashed with temperature gradients from 105 °C to 500 °C in a furnace, and then the ash was dissolved in 2% (*w*/*v*) nitric acid. The sodium was determined by an atomic absorption spectrophotometer (900H, PerkinElmer, Norwalk, CT, USA). The operating conditions were set as the following: wavelength 589 nm, slit width 0.5 nm, lamp current 8 mA, and flow rate of burning gas 1.1 L/min. A standard curve was carried out using the solutions with different sodium content using the same method. The sodium content of marinated beef was obtained according to the vertical value of the standard curve and expressed in g/100 g of the marinated beef.

### 2.4. Sensory Evaluation

Sensory analysis was carried out as described by O’flynn et al. [27] with slight modifications. Eighty unexperienced consumers were invited to form a panel to evaluate the overall acceptability of the marinated beef and eight experienced tasters were invited to form another panel to evaluate six sensory characteristics of them. Prior to being presented to the panelists, the samples were warmed in a water bath (78 °C) until the temperature of the meat center reached 72 °C (about 5 min). The prepared samples of whole cooked marinated beef slices were labeled with individual, three-digit random numbers and served one at a time in a random order. Purified water was provided to panelists for mouth rinsing to clean their palate between sample assessments. The panelists were asked to score the sensory characteristics on a six-point scale including color (1 = very pale, 6 = extremely dark), hardness (1 = very soft, 6 = very firm), juiciness (1 = very dry, 6 = very juicy), saltiness (1 = not salty, 6 = extremely salty), off flavor (1 = not detectable, 6 = extremely intense), overall flavor (1 = not detectable, 6 = extremely intense) and overall acceptability (1 = not acceptable, 6 = extremely acceptable) in separate cabins equipped with daylight illuminant of 1000 lx.

### 2.5. Water Activity Analysis

The water activity (*Aw*) was determined using a water activity meter (HD-3A, Wuxi Huake Instruments and Apparatus Co., Ltd., Wuxi, China) after calibration using saturated sodium chloride solution. One gram of the sample was cut into 3 mm × 3 mm × 3 mm, then put into the sample dish and the chamber was quickly. The response value was reported every 5 min, and the *Aw* value was confirmed when the difference between the two responses was less than 0.005 *Aw*. Each sample was tested in triplicate.

### 2.6. Low-Field Nuclear Magnetic Resonance (LF-NMR)

The moisture composition and distribution of marinated beef was determined according to the method described by Zou Zhang Kang and Zhou [28] with slight modifications. Before making a measurement, all samples were equilibrated for 30 min at 25 °C. The measurements were produced on the NMR analyzing system, MesoMR23-060H-I MRI analyzer (Suzhou Newmai Electronic Technology Co., Ltd., Suzhou, China). The operating field strength was 0.47 T and a corresponding resonance frequency for protons of 22.6 MHz. Transverse relaxation (T_2_) was determined using the Carr–Purcell–Meiboom–Gill (CPMG) sequence with the following parameters: TR = 6500 ms, SW = 200 kHz, D3 = 80 μs, t = 200 μs, NS = 16, and EchoCnt = 10,000, and the attenuation curve was obtained in accordance to the inversion operation using Contin software (the minispec v 1.2). Then, the transverse relaxation times of T_21_ (bound water), T_22_ (immobilized water), T_23_ (free water) and the corresponding peak area ratios P_21_, P_22_ and P_23_ were determined.

### 2.7. Myofibril Fragmentation Index (MFI)

The MFI was determined using a slightly modified version of the procedure used by Kang et al. [29]. Approximately 4 g of marinated beef was added with 40 mL of prechilled solution (100 mmol L^−1^ potassium chloride, 25 mmol L^−1^ dipotassium phosphate, 1 mmol L^−1^ ethylene diamine tetraacetic acid, and 1 mmol L^−1^ magnesium chloride) and homogenized 2 × 30 s with 1 min interval between bursts. The homogenate was centrifuged (Avanti J-E High-Performance Centrifuge, Beckman Coulter Commercial Enterprise (China) Co., Ltd., Shanghai, China) for 15 min at 4 °C, 1000× *g*. The precipitation was resuspended in 40 mL pre-chilled solution and centrifuged again. The final precipitation was resuspended in 20 mL pre-chilled solution. The concentration of the suspension was measured using the Biuret method and then adjusted to 0.5 mg mL^−1^. The absorbance was determined at 540 nm using an ultraviolet spectrophotometer (UV-2000 type, Unico Instrument Co., Ltd., Shanghai, China). The MFI was calculated by multiplying absorbance at 540 nm with 200.

### 2.8. Protein Solubility

Sarcoplasmic protein solubility (SPS) was extracted in 25 mM phosphate buffer (pH 7.2), while total protein solubility (TPS) was extracted in 1.1 M potassium iodide in 0.1 M phosphate buffer (pH 7.2) according to an established procedure [30]. Ground marinated beef (0.25 g) was mixed with the 5 mL buffer and vigorously mixed for 1 min. The mixture was stored for 24 h at 4 °C, then centrifuged for 20 min at 1500× *g*. The protein content of the supernatant was determined by the Lowry method and quantified based on a standard curve prepared from the bovine serum albumin. The myofibrillar protein solubility (MPS) was calculated by TPS minus SPS.

### 2.9. Extraction of Sarcoplasmic and Myofibrillar Proteins and SDS-PAGE

Separation of proteins was performed with the method by Speroni Szerman and Vaudagna [31] with modifications. Adding 0.2 g of chopped marinated beef to 0.8 mL buffer (pH 6.5) with 0.1 M sodium chloride and 0.05 M disodium hydrogen phosphate/sodium dihydrogen phosphate, it was then homogenized at low speed for 1 min and stirred for 20 min at 4 °C. Then, centrifugation was carried out (13,000× *g* for 15 min, 10 °C), the supernatant was the sarcoplasmic protein extract, which was used for sodium dodecyl sulfate-polyacrylamide gel electrophoresis (SDS-PAGE) analysis. The next step of the extraction consisted of exposing the pellet obtained after centrifugation to 0.8 mL buffer (pH 6.5) with 0.6 M sodium chloride and 0.05 M disodium hydrogen phosphate/sodium dihydrogen phosphate. This extraction was also carried out for 20 min at 4 °C followed by a centrifugation in the same conditions indicated above. The supernatants, which contained myofibrillar protein extract, were used for SDS-PAGE analysis.

The obtained sarcoplasmic protein and myofibrillar protein extracts were detected, respectively, using SDS-PAGE gel electrophoresis (5% and 12% polyacrylamide gradient gel). The samples (2 mg mL^−1^) were diluted 1:1 with a Tris–HCl glycerol-containing buffer at pH 6.8 and dissolved in the buffer and denaturized at 96 °C for 10 min. Then, 10 μL of diluted samples were loaded per lane. Electrophoresis was carried out at 100 V. The gel was stained with 0.1% Coomassie Brilliant Blue dye solution. The gel was destained in methanol: acetic acid: H_2_O (5:1:4 by volume). The molecular weight of protein hydrolysate was estimated based on the relative mobility of the protein standard [32].

### 2.10. Scanning Electron Microscopy (SEM)

Referring to the methods of Palka and Daun [33] and Chen, et al. [34] with some modifications. The marinated beef was excised into 3 mm × 3 mm × 10 mm pieces and fixed in 2.5%, pH 7.2 glutaraldehyde solution at room temperature for 2 h. Then the pieces were rinsed with 0.1 M, pH 7.2 phosphoric acid buffer 3 times for 10 min each. Then, the pieces were dehydrated in 30%, 50%, 70%, 80% and 90% ethanol solution for 10 min each time and then absolute ethanol was used (three times). The sample is then lyophilized at 20 Pa, −46 °C using a vacuum freeze dryer (FDU-1200 type, EYELA, Tokyo, Japan). The dried tissue fragments were coated by sputtering and observed with a 10 kV scanning electron microscope. Five photomicrographs of the transverse section were obtained from each sample, and 20 muscle fibers were taken for diameter measurement (Image J software, v 1.8.0), and the average value was taken as the result.

### 2.11. Analysis of Volatile Compounds by Gas Chromatography–Ion mobility Spectrometry Technology (GC-IMS)

Analyses for the identification of characteristic volatile compounds of marinated beef samples were performed on an IMS commercial instrument (FlavourSpec^®^, Dortmund, Germany) fitted with a FS-SE-54-CB-1 quartz capillary column (15 m × 0.5 μm) and an autosampler (CTC Analytics AG, Zwingen, Switzerland). For analysis, the marinated beef sample was heated in a water bath until the center temperature reached 72 °C, and then it was cooled to ambient temperature using running water. One gram of the cooled sample was put in a 20 mL vial that was closed with magnetic cap. After 15 min of incubation (500 g) at 80 °C, 500 μL of sample headspace was automatically infected by means of a heated syringe (85 °C) into the GC injector at 85 °C. After that, the column temperature was maintained at 60 °C, and the nitrogen gas was employed as carrier gas and the flow was programmed based on the following sequence: 2.0 mL min^−1^ for 2 min, gradually increased up to 10 mL min^−1^ in 8 min, up to 100 mL min^−1^ in 10 min and up to 150 mL min^−1^ in 10 min, respectively, for the total running time of 30 min. The drift tube was operated at 45 °C with a 150 mL min^−1^ N_2_ flow. Each analysis was run in triplicate. n-ketones C4~C9 were employed as external references to calculate the retention index (RI) of each compound. All volatile compounds were identified by referencing the RI and the drift time in the GC-IMS library.

### 2.12. Statistical Analysis

The effect of potassium lactate, calcium ascorbate and magnesium chloride as alternative salts on the marinated beef structure and volatile compounds was assessed using the SPSS version 20.0 (SPSS Inc., Chicago, IL, USA). The results were expressed as means and standard error of the mean (SEM). The F-test followed by the post hoc LSD model was conducted at α = 0.05 level, and *p*-value below 0.05 was regarded to be significant. The experiment was repeated three times on different occasions (n = 3). In each replication, the treatment (different pickling solution) was considered as a fixed effect and the replication as a random effect for the four different marinated beef.

## 3. Results and Discussion

### 3.1. Sodium Content

**Figure 1:**
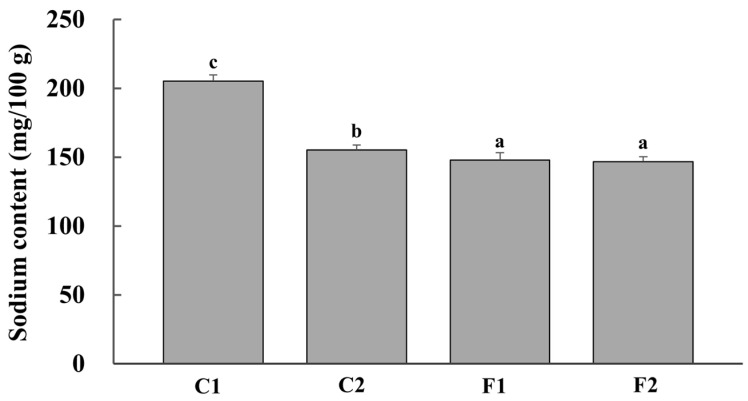
Sodium content of beef slices marinated by full sodium (C1), reductive sodium (C2), and reductive sodium combined with different compound alternative salts (F1 and F2) (SEM7.39). ^a–c^ Means with different superscripts were significantly different at α = 0.05 level.

The sodium contents of beef slices after marinating with different compound substitutions are illustrated in Figure 1. Compared to group C1, the sodium content in all the other groups was much lower due to the decreasing sodium addition, and the reduction rate achieved was 24.39% for group C2, 28.01% for group F1 and 28.49% for group F2, respectively. These rates were much lower than that set for the pickling solution. It may be because of the inadequate penetration of the pickling solution and the presence of a certain amount of sodium in the beef itself. Moreover, the sodium content of group F1 and F2 performed with significant differences to that of group C2. The alternative salt, the only difference between these groups, may be the reason leading to this discrepancy.

### 3.2. Sensory Analysis

The results of sensory evaluation in Table 2 stated that it was difficult for the panelists to decide the group for samples according to their apparent color, although group F1 revealed the greatest mean. In terms of hardness, saltiness and overall flavor, the differences between all the groups were not significant, suggesting that the compound salt substitutions in both groups F1 and F2 basically did not adversely affect the taste perception of the samples. Meanwhile, the juiciness of the marinated beef in all the other groups was significantly lower than that in group C1, which might be related to the reduction in the amount of sodium chloride, leading to more juice loss from the low-sodium beef when curing [23]. In addition, the overall acceptability score of marinated beef in group F1 was similar to that of the control group C1 with no significant difference; however, group C2 and F2 showed similar acceptability with group F1, but they were still not as desirable as group C1 without sodium reduction.

### 3.3. Water Activity

Water activity is an important indicator to describe the moisture content in food. It can be used to indicate the state of moisture, that is, the lower the *Aw* value, the tighter the binding between moisture and other food components, and vice versa. After reducing sodium content, the *Aw* value of samples in all sodium-reduced groups (C2, F1 and F2) increased significantly (*p* < 0.05) compared with the control group C1, indicating that the binding of inner water and muscle fiber in the beef sample may be looser (Table 3). It led to the same opinion with the result previously reported [35], in which they found that the reduction of salt addition (0~2.5%) can significantly increase the water activity in restructured beef products.

### 3.4. LF-NMR

**Figure 2:**
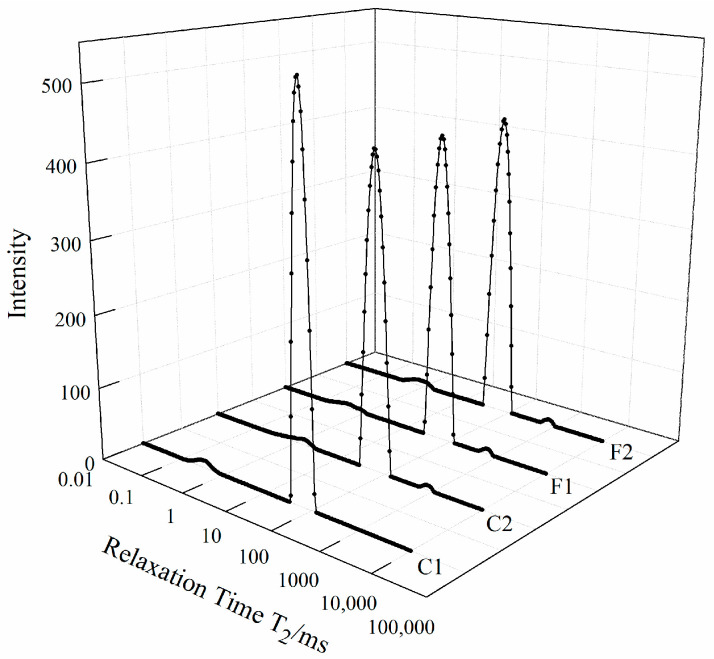
Combined effects of potassium lactate, calcium ascorbate and magnesium chloride as compound alternative salts on the lateral relaxation time, T_2_, in marinated beef with different formulations (C1—full sodium, C2—reductive sodium, F1 and F2 reductive sodium combined with different compound alternative salts).

The effect of compound substitution of salt on the T_2_ relaxation spectrum of marinated beef is shown in Figure 2. The lateral relaxation time, T_2_, can indicate the degree of freedom of moisture in food. Therefore, the relaxation time and relaxation peak area of H protons of the marinated beef samples measured by low-field nuclear magnetic resonance technology (LF-NMR) and multi-exponential fitting, could be used to characterize the fluidity of water in samples of different treatment groups [36]. It can be observed that there were mainly three peaks in marinated beef, representing three types of water closely related to water holding capacity, namely T_2b_ (0.01~10 ms), T_21_ (10~100 ms) and T_22_ (>100 ms). Research has shown that bound water (embodied as T_2b_) refers to the water tightly combined with macromolecular proteins through electrostatic attraction force, accounting for 1% to 5% of the total water content in meat; non-flowing water (embodied as T_21_) exists in the highly organized myofibrillar protein matrix, such as in myofilament, which accounts for more than 90% of the total water in meat [37].

It can be observed in Table 3 that in the four groups of beef samples after different treatments, the third peak in the control group C1 (100% NaCl) disappeared, which represented the relaxation time T_22_, that was free water presenting in myofibrils. This may be because that the fluidity restriction effect of water by Na^+^, and the binding of Cl^−^ to positively charged groups in myofibrillar protein, both resulted in an increased electrostatic repulsion, reduced cohesion, and loose network structure of protein, which contributed to the binding of a large amount of non-flowing water by meat. Therefore, a decrease in salt content can increase the fluidity of water and decrease the water holding capacity [35].

Compared with the T_22_ peak in group C2 (at 689.65 ms), group F1 performed a shorter direction shift to 637.17 ms and F2 performed a longer direction shift to 733.22 ms on the contrary. It indicated that the constraint of free water may have been strengthened or loosed in the muscles with added extra substitutions, and the differences between the alternative salt groups (F1 or F2) and the reduced salt (C2) group were not significant. Therefore, the compound alternative salt had less influence on the free water in beef.

In addition, the P_2b_ values (Table 3) showed a certain fluctuation among the four groups, but the difference was significant between group F2 and the other three, which may be due to the loose binding between protein and the bound water, which was affected by external ions. The ratio of non-flowable water (P_21_) to free water (P_22_) in the muscle was greatly affected by sodium content. Compared with the C1 group, when the sodium salt content decreased, the P_21_ value of group C2 decreased significantly. In group F1, the use of alternative salts can increase the content of non-flowing water to a certain extent and reduce the content of free water. Therefore, the compound substitutions of potassium lactate, calcium ascorbate and magnesium chloride may slow down the water migration, reduce the fluidity, and make up for the loss of moisture caused by salt reduction to a certain extent.

### 3.5. MFI

**Figure 3:**
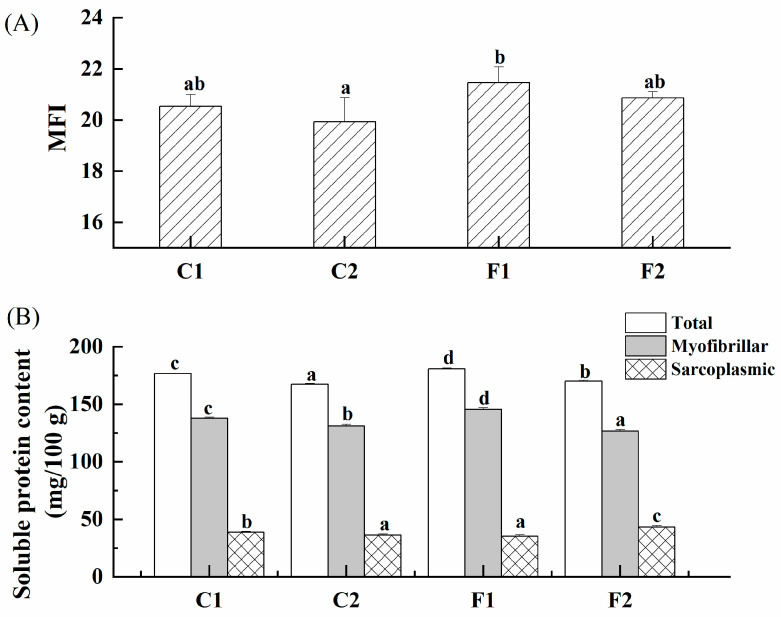
The effects of compound alternative salts on the MFI value ((**A**): SEM0.23) and protein solubility ((**B**): Total, SEM1.36; myofibrillar, SEM0.85; sarcoplasmic, SEM1.86) of marinated beef with different formulations (C1—full sodium, C2—reductive sodium, F1 and F2 reductive sodium combined with different compound alternative salts). Bars and error bars illustrate means and standard deviation, respectively. ^a–d^ Means in different groups with different superscripts were significantly different at α = 0.05 level.

It is generally believed that the tenderness of meat is mainly regulated by the internal sarcomere structure of myofibril, and the tenderness increases when the integrity of the sarcomere structure is destroyed. MFI is an important indicator to characterize the rupture of I-bands and inter-myofibril junctions in muscle cells, reflecting the integrity of muscle fibers and their cytoskeletal proteins [38]. The degree of damage to the internal protein structure of marinated beef can be characterized by the determination of the MFI value. It is considered that the higher the MFI value of meat, the higher the degree of myofibril breakage and the greater the tenderness. As shown in Figure 3A, compared with the control group C1, the MFI in group C2 decreased although not significantly, while that of groups F1 and F2 treated with compound substitutions increased, especially with a higher MFI value for group F1. It implied that alternative salt formulation played an effective role in activity increasing endogenous proteolytic enzymes. In addition, with the calcium ascorbate increasing in beef from group F2, the pH decreased and the environment was further acidified. The activity of endogenous proteolytic enzymes was also reported to be able to be inhibited by the acidic environment, which in turn affected the tenderness of the product [39].

### 3.6. Protein Solubility

The effects of compound substitutions on the protein solubility of marinated beef are shown in Figure 3B. The protein solubility of marinated beef was significantly affected by the compound substitutions (*p* < 0.05). Compared with group C1, the solubility of total soluble proteins (myofibrillar protein and sarcoplasmic protein) in the low-sodium group C2 was significantly decreased (*p* < 0.05). It was mainly because of the salting-in effect in low-salt solution, which was assumed as in the solubility of myofibrillar protein increased with the ionic strength enhancement [40]. While in the substitute group F1, total protein solubility and myofibrillar protein solubility were significantly increased, but myosin solubility was significantly decreased (*p* < 0.05), which may due to the effects of potassium salts and divalent cations on myofibrils [20]. The solubility of protein can promote the extraction of myofibrillar protein, which in turn affect the texture of meat products [41]. In group F2, the ratio of calcium ascorbate and magnesium chloride in the substitution was changed, which seemed to significantly affect the solubility of the protein and performed the opposite variation trend to that in group F1. In previous studies, high concentrations of Ca^2+^ (>5 mM) were found to reduce protein–water interactions by mediating protein–protein interactions, resulting in reduced protein solubility [42] (Pojedinec et al., 2011). It reported that unsalted chicken breasts with lower pH had decreased total protein and myofibrillar protein solubility [43], so the variation of protein solubility in this study may also be responsible to the pH, which inhibited muscle fiber swelling.

### 3.7. SDS-PAGE

**Figure 4:**
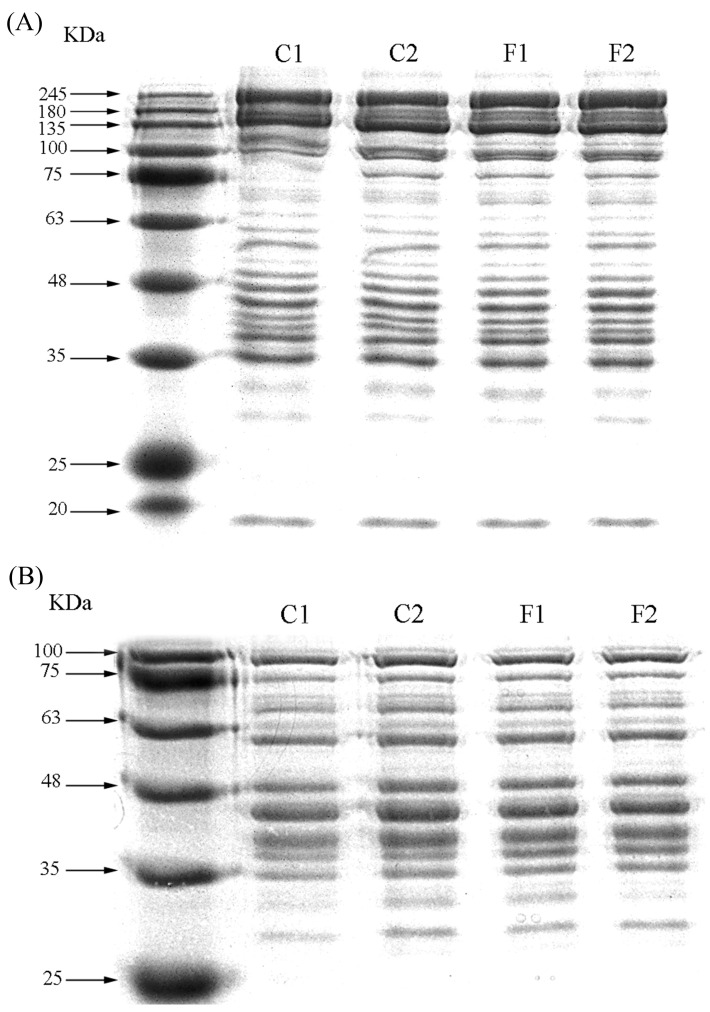
Gel electrophoresis myofibrillar protein (**A**) and sarcoplasmic protein (**B**) of marinated beef with different formulations (C1—full sodium, C2—reductive sodium, F1 and F2 reductive sodium combined with different compound alternative salts).

The electrophoretic pattern of beef myofibril marinated by compound substitutions was illustrated in Figure 4A. Myofibrillar protein is a mixed salt-soluble protein in muscle tissue that plays a vital role in the function of meat products. As can be seen from the figure, its main protein components included the myosin heavy chain (about 200 kDa), paramyosin (about 100 kDa), actin (41~61 kDa), tropomyosin (34~36 kDa), the myosin subunit (35 kDa) and the myosin light chain (16~25 kDa). Similar electropherograms of myofibrillar proteins were also illustrated in a previous study [44]. When the concentration of sodium chloride decreased, the bands of C1 were all found in that of C2, as well as in group F1 and F2, which indicated that the reduced sodium and substitution treatment also caused the dissolution of each protein in the myofibrils.

However, there were some variations on the electrophoresis profile of the marinated beef sarcoplasmic protein shown in Figure 4B. Sarcoplasmic proteins mainly included bands between 30 and 75 kDa. There was little difference in the sarcoplasmic protein profiles between different treatment groups. But it was worth noting that paramyosin and the myosin light chain were observed on the sarcoplasmic protein map. These findings suggested that there was a synergistic effect between NaCl and myofibrillar solubilization, so that part of the myosin released into the soluble fraction [45].

### 3.8. SEM

**Figure 5:**
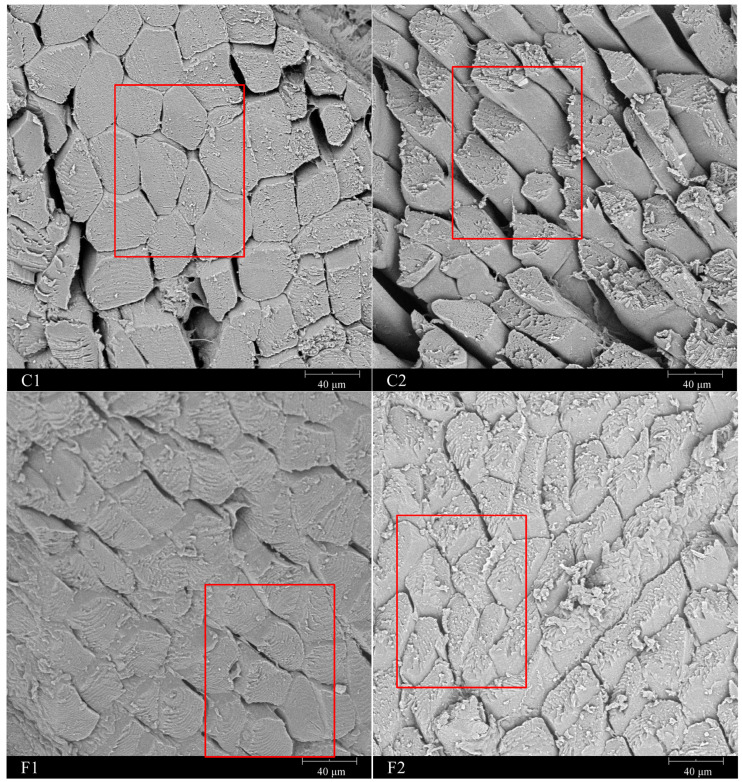
Electron micrographs of muscle fibers of marinated beef with different formulations (C1—full sodium, C2—reductive sodium, F1 and F2 reductive sodium combined with different compound alternative salts) at 1000 magnifications, and the areas in red frames represent the typical structure of muscle fibers for each group.

The effect of compound alternative salts on the surface micromorphology of marinated beef is shown in Figure 5. The cross-section of the muscle fibers was oval, and the displayed microstructures of groups C1 and F1 were similar, in which the structure was relatively complete and the connection between the muscle fibers was tight. But in group C2, the gap between the muscle fibers performed relatively wider due to the direct reduction of sodium chloride, and in group F2, some muscle fibers were elongated and deformed.

Compared with group C1, the muscle fiber diameter of the low-sodium group C2 was significantly reduced (Table 4), which was the smallest among all the groups. Under the effect of salt substitution, the cross-sectional area and diameter of muscle fibers in the F1 and F2 groups increased to some extent, which were 1.85 μm and 0.77 μm higher than those in group C2, respectively, but were still significantly lower than those in group C1. This may be due to the swelling effect of saline solution on muscle fibers. In previous studies, it was found that in 0~0.75 M saline solution, muscle fibers swelling, cross-sectional area increasing, and extracellular space reduction have been observed with the increase in salt ionic strength [46]. Moreover, there was a close relationship between the swelling effect of muscle fibers and the water holding capacity of meat products. In meat products, due to the enhanced binding force between water molecules and muscle proteins, the water holding capacity of the muscles was improved, and the diameter of muscle fibers increased [5].

### 3.9. Volatile Compounds

**Figure 6:**
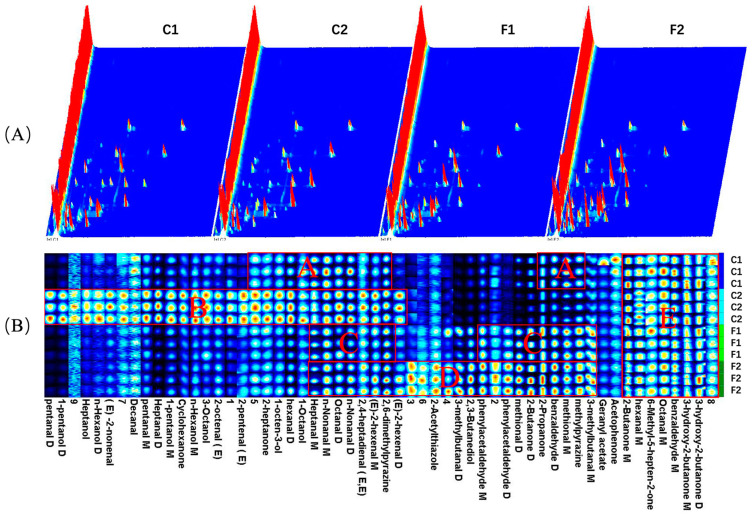
GC-IMS analysis of marinated beef with different formulations (C1—full sodium, C2—reductive sodium, F1 and F2 reductive sodium combined with different compound alternative salts). (**A**) Three-dimensional spectrogram of volatile compounds. Each signal peak represents a volatile compound, and the color represents the concentration of the volatile compound. The color of the dot represents the concentration of the substance, white indicates the same concentration, blue indicates low concentration, and red indicates high concentration; (**B**) fingerprints of volatile compounds. The brighter the spot, the larger the quantity of volatile compounds, and vice versa. Each row represents all the signal peaks selected in a sample. Each column represents the signal peak of the same volatile compounds in different treatments. The regions A–D indicate characteristic peaks for group C1, C2, F1 and F2, and the region E indicates mutual characteristic peak for four groups. The GC-IMS was carried out in triplicate.

Volatile flavor compounds (VFCs) of different formulas of marinated beef were analyzed using headspace GC-IMS. The types of VFCs of the marinated beef at different formulas were similar, but the concentration of VFCs was evidently changed after the sodium chloride substitution treatment due to the ion peak intensities of some VFCs in groups F1 and F2 clearly visualized to be stronger than those in groups C1 and C2 (Figure 6A), indicating that the flavor profile of marinated beef was influenced by the sodium chloride substitution. A total of 33 volatile compounds (monomers and dimers) were identified in all marinated beef samples by Gallery Plot plug-in, including 15 aldehydes, 7 alcohols, 7 ketones, 1 ester, and heterocyclic compounds (1 thiazole and 2 pyrazines) listed in Supplementary Table S1, of which the first three kinds were the main VFCs in all marinated beef samples.

In groups F1 and F2, contents of the flavor substances of benzaldehyde, 3-methylbutyraldehyde, and 3-methylthiopropanal, belonging to Strecker aldehydes, performed significantly higher than those in group C1, and the lowest content of them was in group C2. These three compounds were reported to be formed by the degradation of their amino acid precursors [47]. In some previous studies, it was found that the use of potassium, magnesium and calcium salts could increase the activity of protein hydrolase and aminopeptidase to some extent, promoting the degradation of macromolecular proteins and generating more free amino acids, which contributed to the flavor of cured meat products [48,49]. It was also confirmed in the results of this study. Meanwhile, the content of ketones in the beef samples was also the lowest in group C2 and the highest in group F2, with significant differences between them; only their contribution to flavor was relatively less compared to aldehydes [50]. However, 1-octen-3-ol with a low threshold concentration revealed a higher concentration in the C2 group (Figure 6B), which may be related to the activity of microorganisms. Combining with Table 1, it can be seen that *Aw* increased as the sodium salt content decreased, and the bacteriostatic effect may weaken, which in turn promoted the action of microbial enzymes to degrade branched-chain aldehydes into related branched-chain alcohols [51].

The results of previous studies on the effect of alternative salt on VFCs in meat products varied widely. A study by Armenteros et al. [3] has found that the processing of dry-cured ham produced higher amounts of volatile compounds, and the ham from Recipe I (100% sodium chloride) had a higher content of lipid oxidative derivatives (such as hexanal, nonanal) than that from Recipe III (55% sodium chloride, 25% potassium chloride, 15% calcium chloride and 5% magnesium chloride), while the latter had a significantly higher content of Strecker aldehydes (such as 3-methylbutanal) and ketones. It was also found in another study that there was no significant difference in acid lipase activity and lipid oxidation between the substitution group (formulation of sodium chloride, calcium chloride and magnesium chloride) and the control group (100% sodium chloride), with an increase in lipid hydrolysis [52]. This difference in results will be bound to induce manifold VFCs mappings, and it may be related to the large differences in the types of processed meat products, processing methods and the composition of compound substitutions.

## 4. Conclusions

The alternative salt formulation was feasible to partially replace the conventional sodium chloride in the marinated beef slice product, and the formulation of group F1 showed the best effect on the comprehensive quality characteristics. The sodium content can decrease up to 28% in all test groups than that in the full sodium group, C1. Better tenderness and water retention capacity and higher release of volatile flavor compounds were found in the sample of alternative groups, F1 and F2. The moisture distribution, microstructure and overall sensory attributes of group F1 were more similar to those of group C1, and it even demonstrated better flavor characteristics. The results suggested that F1 formulation might be applicable in low-sodium marinated beef production. However, future research should focus on the influence of alternative salts on the processing and quality of marinated meat during the vacuum tumbling period, which will have practical implications for industrial production.

## Figures and Tables

**Table 1:** Percentage of sodium chloride and three substitutions in pickling liquid for different treatment groups. The 4% (*w*/*w*) sodium chloride in group C1 was denoted as 100%, and the salt substitutions of other groups, C2, F1 and F2 were converted proportionally according to the additional amount of group C1. “-” means no addition.

Groups	Sodium Chloride/%	Potassium Lactate/%	Calcium Ascorbate/%	Magnesium Chloride/%
C1	100	-	-	-
C2	60	-	-	-
F1	60	20	10	10
F2	60	20	15	5

**Table 2:** The effects of compound alternative salts on the sensory evaluation of marinated beef with different formulations (C1—full sodium, C2—reductive sodium, F1 and F2 reductive sodium combined with different compound alternative salts).

Groups	Color	Hardness	Juiciness	Saltiness	Off Flavor	Overall Flavor	Overall Acceptability
C1	3.25 ^a^	2.75 ^a^	4.25 ^b^	3.50 ^a^	1.25 ^a^	4.25 ^a^	5.00 ^b^
C2	3.50 ^a^	2.88 ^a^	2.75 ^a^	3.50 ^a^	2.00 ^a^	3.75 ^a^	3.75 ^a^
F1	3.88 ^a^	3.75 ^a^	2.75 ^a^	3.50 ^a^	1.50 ^a^	4.00 ^a^	4.50 ^ab^
F2	2.75 ^a^	3.25 ^a^	2.75 ^a^	2.75 ^a^	1.00 ^a^	3.50 ^a^	3.75 ^a^
SEM	0.36	0.83	0.35	0.64	0.40	0.32	0.41
*p*-value	0.055	0.634	0.002	0.574	0.126	0.168	0.025

^a,b^ Means in the same column with different superscripts were significantly different at α = 0.05 level.

**Table 3:** The effect of compound alternative salts on the *Aw*, transverse relaxation time (T_2_) and peak area ratio (P_2_) in marinated beef with different formulations (C1—full sodium, C2—reductive sodium, F1 and F2 reductive sodium combined with different compound alternative salts).

Groups	*Aw*	T_2_/ms			P_2_/%		
		T_2b_	T_21_	T_22_	P_2b_	P_21_	P_22_
C1	0.931 ^a^	0.45 ^a^	69.09 ^b^	-	2.25 ^a^	97.75 ^b^	-
C2	0.938 ^bc^	1.26 ^b^	57.42 ^a^	689.65 ^ab^	2.27 ^ab^	96.55 ^a^	1.18 ^a^
F1	0.936 ^b^	0.51 ^a^	54.79 ^a^	637.17 ^a^	2.23 ^a^	96.79 ^a^	0.98 ^a^
F2	0.940 ^c^	0.93 ^b^	53.56 ^a^	733.22 ^b^	2.44 ^b^	96.80 ^a^	0.76 ^a^
SEM	0.001	0.09	1.71	26.40	0.05	0.14	0.10
*p*-value	0.001	0.001	0.000	0.112	0.084	0.000	0.118

^a–c^ Means in the same column with different superscripts were significantly different at α = 0.05 level. “-” Means undetected.

**Table 4:** Muscle fiber diameters of marinated beef with different formulations (C1—full sodium, C2—reductive sodium, F1 and F2 reductive sodium combined with different compound alternative salts).

Groups	Muscle Fiber Diameter/μm
C1	37.73 ^d^
C2	35.53 ^a^
F1	37.38 ^c^
F2	36.30 ^b^
SEM	0.23
*p*-value	0.000

^a–d^ Means with different superscripts were significantly different at α = 0.05 level.

## Data Availability

The original contributions presented in the study are included in the article/Supplementary Material, further inquiries can be directed to the corresponding author.

## Supplementary Materials

The following supporting information can be downloaded at: https://www.mdpi.com/article/10.3390/foods13020291/s1, Supplementary Table S1. Qualitative analysis information of volatile flavor substances in marinated beef. Values represent the mean ± S.E. (n = 3). The upper lowercase letters indicate significantly different in the same column (*p* < 0.05). MW: molecular weight; RI: retention index; RT: retention time; DT: drift time; volume (a.u.): peak volume.
