# Supplementary material for: Comprehensive Effects of Potassium Lactate, Calcium Ascorbate and Magnesium Chloride as Alternative Salts on Physicochemical Properties, Sensory Characteristics and Volatile Compounds in Low-Sodium Marinated Beef
Source: Foods. 2024 Jan 17;13(2):291. doi: 10.3390/foods13020291 (PMC10814945; doi:10.3390/foods13020291)
Supplement: Supplementary file 1 [file foods-13-00291-s001.zip › foods-2730656-supplementary.pdf]

Supplementary Table S1 Qualitative analysis information of volatile flavor substances in marinated beef. Values represent the mean ± S.E. (n = 3). The upper lowercase letters indicate significantly different in the same column ($P < 0.05$). MW: molecular weight; RI: retention index; RT: retention time; DT: drift time; volume (a.u.): peak volume.

No.	Compound	CAS#	Formula	MW	RI	Rt(s)	Dt(a.u.)	volume (a.u.)			
								C1	C2	F1	F2
**Ketones**											
1	2-Propanone	C67641	C_3H_6O	58.1	513.0	113.467	1.12392	4351.12±215.50^{b}	3385.46±118.98^{a}	6054.56±437.92^{c}	7552.66±291.32^{d}
2	2-Butanone-M	C78933	C_4H_8O	72.1	575.3	140.698	1.06685	2482.01±129.67^{a}	2396.42±154.02^{a}	2727.76±331.46^{a}	2691.47±71.65^{a}
3	2-Butanone-D	C78933	C_4H_8O	72.1	577.7	141.726	1.25239	2296.98±135.00^{a}	1390.78±3.80^{a}	4080.28±908.40^{b}	6063.9±795.73^{c}
4	3-Hydroxy-2-butanone-	C513860	$C_4H_8O_2$	88.1	705.0	203.633	1.05839	4228.5±265.12^{a}	4230.24±44.43^{a}	3816.85±266.36^{ab}	3663.44±411.55^{a}
5	3-Hydroxy-2-butanone-D	C513860	$C_4H_8O_2$	88.1	709.4	207.230	1.33182	7132.57±508.94^{ab}	6472.24±161.23^{a}	7574.95±109.87^{ab}	7851.79±1002.29^{b}
6	2-Heptanone	C110430	$C_7H_{14}O$	114.2	892.9	393.972	1.26498	127.76±22.32^{a}	184.79±3.06^{b}	126.34±19.38^{a}	135.07±4.29^{a}
7	Cyclohexanone	C108941	$C_6H_{10}O$	98.1	896.9	401.310	1.15173	159.91±20.01^{a}	334.57±13.61^{b}	159.84±19.83^{a}	163.18±5.61^{a}
8	6-Methyl-5-hepten-2-one	C110930	$C_8H_{14}O$	126.2	991.4	577.665	1.18050	177.07±33.51^{a}	159.39±7.79^{a}	164.44±17.12^{a}	151.38±13.51^{a}
9	Acetophenone	C98862	C_8H_8O	120.2	1065.2	714.661	1.18378	122.72±21.42^{b}	94.41±4.59^{a}	80.9±2.42^{a}	84.35±6.15^{a}
10	3-Methylbutanal-M	C590863	$C_5H_{10}O$	86.1	641.0	169.373	1.17608	1113.96±79.25^{a}	1140.58±151.97^{a}	1809.38±154.23^{b}	1896.01±124.35^{b}
11	3-Methylbutanal-D	C590863	$C_5H_{10}O$	86.1	643.2	170.346	1.40841	225.72±14.16^{a}	231.29±45.84^{a}	1071.7±529.37^{b}	1933.54±342.21^{c}
12	Pentanal-M	C110623	$C_5H_{10}O$	86.1	691.6	192.558	1.18273	527.52±36.02^{b}	1049.06±37.32^{c}	488.66±50.21^{ab}	426.73±36.07^{a}
13	Pentanal-D	C110623	$C_5H_{10}O$	86.1	691.2	192.331	1.42426	56.01±14.73^{a}	464.14±57.18^{b}	65.77±19.03^{a}	61.92±14.24^{a}
14	2-Pentenal-(E)	C1576870	C_5H_8O	84.1	745.1	236.461	1.10614	42.71±6.68^{a}	152.65±15.58^{c}	66.09±13.22^{b}	79.09±12.12^{b}
15	Hexana-M	C66251	$C_6H_{12}O$	100.2	791.0	276.330	1.25434	1013.34±18.93^{a}	1028.37±74.46^{a}	978.56±51.86^{a}	1006.77±82.32^{a}

Supplementary Table S1 (Continued)

No.	Compound	CAS#	Formula	MW	Rl	Rt(s)	Dt(a.u.)	Volumn(a.u.)			
								C1	C2	F1	F2
16	Hexanal-D	C66251	$C_6H_{12}O$	100.2	791.2	276.586	1.56423	1702.81±349.83^{a}	3769.32±139.04^{b}	1525.43±267.45^{a}	1799.96±258.03^{a}
**Aldehydes**											
17	(E)-2-Hexenal-M	C6728263	$C_6H_{10}O$	98.1	846.7	340.325	1.18086	200.13±38.50^{a}	333.96±22.04^{c}	225.39±26.13^{ab}	285.12±46.23^{bc}
18	(E)-2-Hexenal-D	C6728263	$C_6H_{10}O$	98.1	846.7	340.325	1.51962	33.31±9.31^{a}	85.27±8.33^{b}	46.86±9.47^{a}	68.71±14.17^{b}
19	Heptana-M	C111717	$C_7H_{14}O$	114.2	900.2	407.457	1.33354	938.6±106.10^{b}	1163.07±44.23^{c}	743.39±30.98^{a}	698.11±22.66^{a}
20	Heptanal-D	C111717	$C_7H_{14}O$	114.2	900.2	407.457	1.69918	700.01±128.70^{b}	2239.8±101.91^{c}	485.4±53.59^{a}	437.49±47.04^{a}
21	Methional-M	C3268493	C_4H_8OS	104.2	903.8	414.190	1.09308	1218.84±52.05^{b}	663.13±15.65^{a}	1624.23±25.58^{c}	1792.84±77.33^{d}
22	Methional-D	C3268493	C_4H_8OS	104.2	902.8	412.492	1.40093	1208.4±233.64^{b}	616.36±23.70^{a}	3076.61±75.52^{c}	4374.35±231.99^{d}
23	Benzaldehyde-M	C100527	C_7H_6O	106.1	953.9	507.675	1.15072	1926.59±157.82^{b}	1646.58±15.66^{a}	1940.35±29.30^{b}	2030.4±113.74^{b}
24	Benzaldehyde-D	C100527	C_7H_6O	106.1	955.9	511.590	1.47186	2957.13±292.77^{b}	1912.95±32.66^{a}	3820.89±189.79^{c}	4886.47±133.40^{d}
25	Octanal-M	C124130	$C_8H_{16}O$	128.2	1011	614.158	1.40222	1705.75±172.47^{bc}	1861.99±19.44^{c}	1453.23±153.13^{a}	1602.13±24.01^{ab}
26	Octanal-D	C124130	$C_8H_{16}O$	128.2	1008.9	610.243	1.82590	1596.11±207.20^{a}	2469.06±264.26^{b}	1218.38±453.60^{a}	1568.62±129.01^{a}
27	2,4-Heptadienal-(E,E)	C4313035	$C_7H_{10}O$	110.2	1020.5	631.797	1.18958	138.97±27.11^{a}	181.82±22.45^{b}	141.33±5.86^{a}	156.77±12.81^{ab}
28	Phenylacetaldehyde-M	C122781	C_8H_8O	120.2	1045.3	677.761	1.25044	407.67±11.47^{b}	323.1±13.99^{a}	854.06±25.29^{c}	1040.17±17.90^{d}
29	Phenylacetaldehyde-D	C122781	C_8H_8O	120.2	1046.0	679.056	1.53882	36.79±1.71^{a}	32.84±2.77^{a}	112.71±8.82^{b}	165.26±0.44^{c}
30	2-Octenal-(E)	C2548870	$C_8H_{14}O$	126.2	1062.9	710.365	1.33702	164.02±23.62^{a}	312.98±12.53^{c}	188.06±15.26^{a}	241.82±7.51^{b}
31	n-Nonana- M	C124196	$C_9H_{18}O$	142.2	1106.5	791.308	1.47388	2721.63±323.61^{ab}	2872.05±107.35^{b}	2416.52±42.19^{a}	2653.94±104.10^{ab}
32	n-Nonanal-D	C124196	$C_9H_{18}O$	142.2	1106.5	791.308	1.94471	1912.17±469.76^{ab}	2353.09±332.55^{b}	1490.17±141.93^{a}	2061.02±350.93^{ab}
33	(E)-2-Nonenal	C18829566	$C_9H_{16}O$	140.2	1166.3	902.085	1.41436	162.55±44.35^{a}	414.94±46.42^{b}	185.23±15.52^{a}	183.42±12.20^{a}

Supplementary Table S1 (Continued)

No.	Compound	CAS#	Formula	MW	Rl	Rt(s)	Dt(a.u.)	Volumn(a.u.)			
								C1	C2	F1	F2
34	Decanal	C112312	$C_{10}H_{20}O$	156.3	1222.4	1006.133	1.54092	195.45±34.25[a]	205.53±11.72[a]	174.01±10.53[a]	178.89±2.86[a]
**Alcohols**											
35	1-Pentanol-M	C71410	$C_5H_{12}O$	88.1	761.6	250.073	1.24957	499.79±31.02[a]	1097.92±56.44[b]	460.85±81.87[a]	423.47±64.31[a]
36	1-Pentanol-D	C71410	$C_5H_{12}O$	88.1	760.5	249.101	1.51036	77.96±11.16[a]	393.38±40.17[b]	68.94±10.13[a]	64.44±16.15[a]
37	2,3-Butanediol	C513859	$C_4H_{10}O_2$	90.1	777.7	263.199	1.36574	538.02±36.54[b]	374.05±24.98[a]	1419.66±72.16[c]	2282.75±137.60[d]
38	n-Hexanol-M	C111273	$C_6H_{14}O$	102.2	871.8	369.192	1.32675	258.59±24.13[a]	491.93±31.46[b]	230.18±2.95[a]	253.83±8.31[a]
39	n-Hexanol-D	C111273	$C_6H_{14}O$	102.2	869.6	366.645	1.6383	23.9±2.69[a]	53.81±7.34[b]	21.24±3.00[a]	25.83±4.74[a]
40	Hheptanol	C53535334	$C_7H_{16}O$	116.2	976.7	550.349	1.39819	91.37±15.86[a]	243.9±23.11[b]	83.81±11.33[a]	77.95±0.81[a]
41	1-Octen-3-ol	C3391864	$C_8H_{16}O$	128.2	984.3	564.471	1.16060	523.64±53.36[b]	768.41±19.97[c]	410.92±66.88[a]	521.42±35.70[b]
42	3-Octanol	C589980	$C_8H_{18}O$	130.2	986.3	568.301	1.39959	174.24±19.92[a]	344.15±12.37[b]	181.39±42.69[a]	143.1±22.24[a]
43	1-Octanol	C111875	$C_8H_{18}O$	130.2	1081.6	745.048	1.47061	294.76±56.11[ab]	349.18±40.63[b]	267.46±36.21[a]	267.94±9.86[a]
**Esters**											
44	Geranyl acetate	C105873	$C_{12}H_{20}O_2$	196.3	1346.5	1236.389	1.22031	856.25±325.32[a]	669.29±28.28[a]	654.88±20.04[a]	708.44±52.94[a]
**Heterocycles**											
45	Methylpyrazine	C109080	$C_5H_6N_2$	94.1	779.4	264.658	1.08954	1693.35±83.86[a]	2005.23±39.22[b]	2275.66±119.53[c]	2531.98±180.90[d]
46	2,6-Dimethylpyrazine	C108509	$C_6H_8N_2$	108.1	900.1	407.362	1.53854	576.55±14.31[b]	461.07±25.18[a]	876.1±37.99[c]	1017.18±86.01[d]
47	2-Acetylthiazole	C24295032	C_5H_5NOS	127.2	1023	636.356	1.13140	1068.84±78.50[a]	1490.49±18.87[b]	1342.62±106.15[b]	1436.01±111.66[b]
